# Microstructural and Thermo-Optical Properties of Cassava and Gellan Gum Films: A Photoacoustic Study

**DOI:** 10.3390/polym18030313

**Published:** 2026-01-23

**Authors:** Ámbar Belén Ortega-Rubio, José Abraham Balderas-López, Mónica Rosalía Jaime-Fonseca

**Affiliations:** 1 Departamento de Ingeniería Bioquímica, Escuela Nacional de Ciencias Biológicas, Instituto Politécnico Nacional, Gustavo A. Madero, Mexico City 07738, Mexico; 2 Unidad Profesional Interdisciplinaria de Biotecnología, Instituto Politécnico Nacional, Av. Acueducto S/N, Col. Barrio la Laguna, Ticomán, Mexico City 07340, Mexico; 3 Centro de Investigación en Ciencia Aplicada y Tecnología Avanzada Legaria, Instituto Politécnico Nacional, Av. Legaria 694, Col. Irrigación, Mexico City 11500, Mexico; mjaimef@ipn.mx

**Keywords:** biodegradable film, aniline, photoacoustics, gellan gum, thermal diffusivity, optical properties

## Abstract

The growing global production of plastic, which reached 460 million tonnes in 2022 and has projections of 5.4 million tonnes of waste by 2050 without intervention, has created a severe environmental crisis that demands the development of sustainable alternatives. In this context, this study aims to characterise biodegradable films based on cassava starch and gellan gum, combining microstructural and mechanical properties with the evaluation of thermo-optical parameters. An important advance was the pioneering application of a self-normalised photoacoustic technique, used for the first time to measure thermal diffusivity (0.0013 ± 0.0002 cm^2^/s) and optical absorption coefficients (at 660 nm) as a function of different concentrations of aniline blue. The results validate the material, which showed high solubility (89.23 ± 1.03%) and crystallinity of 27.40 ± 1.68%. The film demonstrated remarkable biodegradability, losing almost all of its weight (98.30 ± 1.01%) in just 15 days. The measurement of the optical absorption coefficients (at 660 nm) confirmed a linear relationship with the concentration of aniline, validating Beer–Lambert’s law and providing the absorptivity of the dye within the solid matrix—something inaccessible with conventional methods. In conclusion, these films offer significant potential as a viable ecological substitute for single-use plastics, contributing significantly to mitigating the global impact of plastic waste.

## 1. Introduction

The increase in global plastic production and consumption, which reached 460 million tonnes in 2022, with almost 40% of this amount used for packaging due to its flexibility, strength, and low cost, has resulted in serious environmental problems. In a scenario without change, it is estimated that plastic waste generation will reach 2.5, 3.7, and 5.4 million tonnes in 2030, 2040, and 2050, respectively. This circumstance is promoting an urgent search for sustainable alternatives, among which biodegradable plastics stand out [1,2].

Bioplastics are divided into two categories, biodegradable and non-biodegradable, regardless of their origin.

Plastics that are biodegradable include ester and amide bonds, which facilitate their total decomposition through aqueous and enzymatic hydrolysis processes, generating CH_4_, CO_2_, and H_2_O as end products, without the creation of toxic substances. Their degradation cycle is considerably shorter compared to traditional polymers, which take more than a century to decompose [3]. The general characteristics of biodegradable plastics are detailed in Table 1.

It is important to note that the close relationship between the composition of polymers and their physicochemical properties (mechanical, optical, thermal, and chemical) is essential for the formulation of specific materials. Structural elements such as chemical composition, molecular weight, structural order (crystallinity), cross-linking, and intermolecular interactions (such as van der Waals forces and hydrogen bonds) are decisive in fundamental characteristics such as glass transition temperature (Tg) and melting temperature (Tf). For example, greater chain rigidity leads to an increase in Tg and Tf temperatures [4].

This dependence on structural characteristics is vital for categorising the mechanical behaviour of polymers (whether fibres, plastics, or elastomers) and estimating their performance during stress and strain tests. Based on their processing capabilities, polymers are classified into two main groups:

- Thermosets: These are engineering polymers, such as phenolic resins or polyurethanes, characterised by their cross-linked (strongly intertwined) structure. This arrangement gives these materials remarkable mechanical strength, thermal stability, and durability against corrosion, although it limits their ability to be modified or moulded after formation [2].
- Thermoplastics: Examples of these are polyethylene (PE), polypropylene (PP), polyvinyl chloride (PVC), and polyethylene terephthalate (PET), which consist of linear, flexible chains. The lack of rigid cross-links allows for these materials to be easily heated, moulded, or altered [2].

On the other hand, non-biodegradable plastics, such as polyethylene terephthalate (PET) and polypropylene (PP), are petroleum products and remain in the environment, making their recycling and reuse essential to reducing environmental impact.

Furthermore, to achieve specific properties, these plastics often require the addition of compounds (such as plasticisers and stabilisers) because without them they tend to show greater degradability and inferior thermal, mechanical, and physical properties [5].

While the advancement of plastics has made human life easier, it has also generated a large volume of hazardous waste that poses threats to the environment and living beings. Plastic items (including bottles, bags, containers, packaging materials, and accessories) constitute a significant portion (about 12%) of the total inorganic content in municipal solid waste [6]. Single-use plastic products (such as polyethylene bags and food utensils) are estimated to contribute approximately 40% of this waste, which ends up in landfills or the marine environment [7].

**Figure 1:**
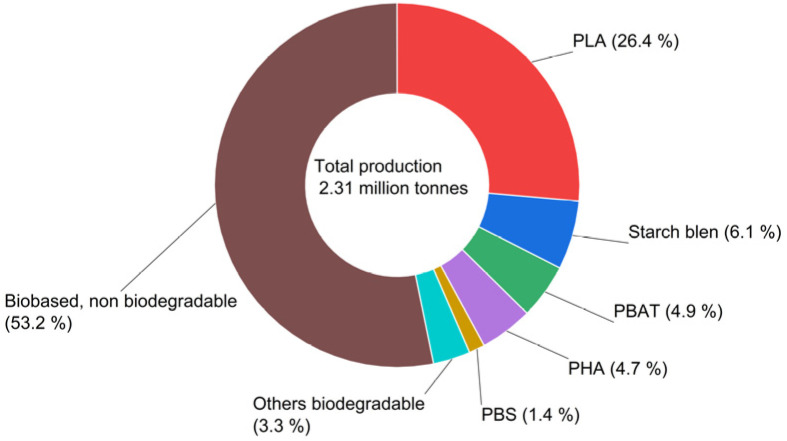
Global bioplastics production capacity in 2025. PLA: polylactic acid; PHA: polyhydroxyalkanoate; PBS: polybutylene succinate; PBAT: polybutylene adipate terephthalate (Source: European Bioplastics e. V [8]).

In 2025, global bioplastic production capacity reached 2.31 million tonnes, divided between non-biodegradable (53.2%) and biodegradable (46.8%) polymers (see Figure 1). The packaging sector remains the main consumer, using 41.3% (0.95 million tonnes) of total capacity [8].

Beyond packaging, bioplastics have diversified into various fields, including textiles, consumer goods, and agriculture. Their applications range from eugenol microencapsulation [9] and packaging for microbial protection of food [10] to microneedle patches for pharmaceutical delivery [11] and studies to characterise properties such as thermal diffusivity in nanocomposite films [12].

Despite this expansion, the market share of bioplastics remains below 1% of the total plastics industry, which is significantly lower than overall market demand [13].

The large gap between bioplastics production and market demand highlights the complexity of the problem. This underscores the need to research and improve the end-of-life management of these materials, seeking methods to obtain added value before disposal, thereby mitigating the environmental impact of polymer waste [14]. Consequently, replacing conventional plastic with biodegradable bio-based plastics (such as starch-based plastics) can substantially reduce the risk of hazardous chemicals and mitigate the effects of climate change due to their lower carbon footprint [15].

For this reason, cassava starch is a preferred option due to its lower amylose content compared to potato starch, which facilitates the formation of softer gels and viscous solutions, ideal for biodegradable films [16,17]. Gellan gum, a polysaccharide produced by *Pseudomonas elodea*, is incorporated to improve the strength and flexibility of polymer networks, thus optimising the mechanical properties of the resulting polymers [18,19].

In addition, low-molecular-weight plasticisers, such as glycerol, are used to prevent crystallisation and increase the flexibility of the polymer matrix.

Edible and biodegradable films, such as those based on cassava starch, have emerged as a key technological trend, driven by the need for sustainable packaging solutions that promote economic growth.

Cassava starch is a promising material due to its ability to be a renewable, biodegradable, transparent, non-toxic source with good film-forming capacity, as well as offering an effective barrier against oxygen. For cassava starch to achieve competitive commercial application, research has focused on improving its mechanical and barrier properties, which are critical for extending the shelf life of food. Key strategies include chemical modification of the starch and the incorporation of additives, such as plasticisers (glycerol), blending with other biopolymers, or the use of gums [20].

Gellan gum (GG) films and coatings have established themselves as biodegradable and environmentally friendly alternatives to petrochemical plastic in food packaging, with their main application being in active and intelligent packaging to preserve quality and extend the shelf life of perishable products. These films are modified with additives such as essential oils, plant extracts, and nanoparticles to enhance crucial properties such as light barriers, antioxidant activity, and antimicrobial capabilities. GG is used effectively to coat fruits and vegetables (apples, blueberries, strawberries), reducing weight loss, respiration rate, and microbial deterioration, which can extend their freshness by up to 56 days in the case of blueberries. They are also essential for meat and fish products (pork, chicken, prawns), as they inhibit lipid oxidation and the growth of microorganisms, extending shelf life in refrigeration and freezing. In addition, the incorporation of natural pigments allows them to act as visual indicators of freshness, facilitating real-time monitoring of deterioration, consolidating GG as an innovative and sustainable packaging solution [21].

The characterisation of the biodegradable polymers mentioned in previous paragraphs is traditionally carried out using mechanical, optical, and structural tests, including techniques such as Fourier transform infrared spectroscopy (FTIR) [22]. However, photoacoustic techniques, being non-invasive and economical, offer a significant advantage: they allow for the simultaneous measurement of thermal diffusivity and optical absorption coefficient at a specific wavelength. These properties, crucial for understanding thermal and optical behaviour, are rarely explored in biodegradable films using conventional methods.

To measure the thermal diffusivity of solids, the laser flash method is the most widely used conventional transient technique, valued for its high reliability and ISO standardisation. However, it requires developing the sample to a specific size (a disc 10 mm in diameter and 1–2 mm thick) and measuring the thermal response on the back side, which makes non-destructive measurements difficult [23].

In contrast, the photoacoustic (PA) method, based on the basic one-dimensional Rosencwaig and Gersho (RG) theory, offers a key advantage: it allows for non-destructive, single-sided measurement of the material in its original state. The RG theory models the modulated light absorption in the sample, generating acoustic waves (PA signal) that depend on thermal properties. Its applications have included the measurement of thermal properties of thin films, coatings, and multilayer materials, and it has been used for in vivo evaluation of skin [23].

A fundamental aspect of Rosencwaig and Gersho (RG) theory is the concept of thermal diffusion length, which indicates the effective depth in the material at which the photothermal signal is generated. This depends on the thermal diffusivity of the material, as well as the modulation frequency, and must be less than the thickness of the material under study for the RG model to be applied effectively. Samples that satisfy this condition in the frequency range under study are called thermally thick. This condition applies very well to thin polymer samples, given the low thermal diffusivity of these materials, as reported by Fernández-Olaya et al. [24]. Therefore, PA techniques are ideal for characterising biodegradable thin films.

On the other hand, today Beer–Lambert’s Law in UV-Vis spectroscopy relates the absorbance (A) of a solution to its concentration (c) and the length of the path (l) of light through it, using the expression *A* = *ϵ*l*c*, where *ϵ* is the molar absorptivity, an intrinsic optical property of the substance in the solution. This law is fundamental for determining unknown concentrations by measuring how much monochromatic light a sample absorbs, since absorption is directly proportional to the molecules present. However, this method is strictly valid for dilute solutions, generally less than 0.01 M (10 mM). At higher concentrations, the average distance between solute molecules decreases, increasing electrostatic and near-field interactions between them [25]. These interactions alter the molar absorptivity of the analyte, causing deviations from linearity (curvature in the calibration graph), so it is not possible to use this method to appreciate a linear correlation when using different concentrations of a dye in biodegradable films.

Therefore, the fundamental purpose of this research was to analyse biodegradable films composed of cassava starch and gellan gum, adding glycerol to improve their mechanical characteristics. The aim was to establish a linear relationship between the optical properties and the concentration of a dye (blue aniline), as well as to assess the thermal diffusivity in these polymer films. Finally, the objective was to relate these optical and thermal properties to the microstructural, mechanical, and physical qualities of the material.

The characterisation was carried out by combining conventional methods, such as X-ray diffraction (XRD) and mechanical testing, with a novel self-normalised photoacoustic technique. The latter was used to determine the thermal diffusivity and absorption coefficient (at 660 nm) of the films at various concentrations of blue aniline. This dye was selected for its photostability to light at the wavelength used, a fundamental condition for the accuracy of the measurements made with this technique.

The result of this research was the creation and characterisation of a translucent, semi-crystalline biodegradable film with significant water solubility, which gives it high biodegradability. For the first time, the measurement of the optical absorption coefficients at 660 nm in the films with the dye confirmed a linear relationship with the concentration of aniline, validating Beer–Lambert’s law. In addition, rapid biological degradation was demonstrated, with a weight reduction of 98.30 ± 1.01% in just 15 days in a natural environment, highlighting its remarkable potential as a sustainable and environmentally friendly plastic.

## 2. Materials and Methods

### 2.1. Materials

Blue aniline dye was purchased from ANYLQUIMTEX (Monterrey, Mexico). Glycerol, deionised water, acetic acid (99.7%), and sodium bicarbonate were purchased from Merck KGaA (Darmstadt, Germany). All reagents were analytical grade, except for gellan gum, which was purchased from MCS Molecular Cuisine Supplies (Puebla, Mexico), and it was molecular culinary grade. Cassava starch was purchased from Erawan Marketing Co. (Bangkoknoi, Thailand).

### 2.2. Methods

#### 2.2.1. Preparation of the Base Solution for Films

Two formulations were developed and combined to obtain films with optimal characteristics. For the first formulation, 100 mL of water was heated to 80 °C, then gellan gum was gradually added under mechanical stirring at 550 rpm until a 2% (*w*/*v*) gellan gum suspension was obtained. The process took about 20 min until homogenisation was achieved.

**Figure 2:**
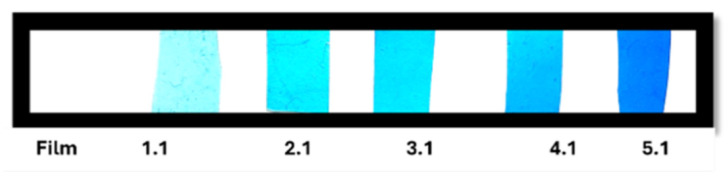
Cassava starch films and gellan gum at different concentrations of blue aniline.

**Figure 3:**
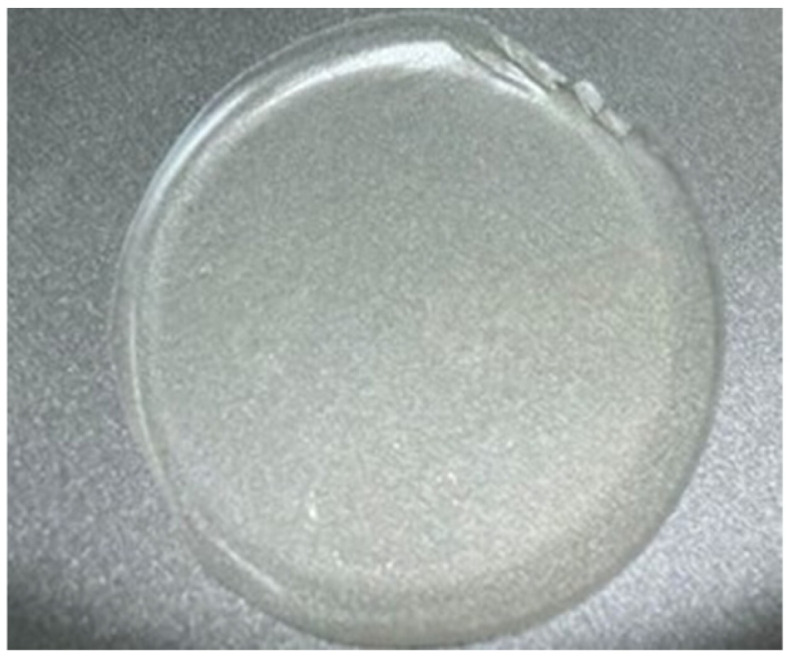
Films containing 2% (*w*/*v*) gellan gum, 10% (*w*/*w*) glycerol, 2% (*w*/*v*) cassava starch, and 1% (*w*/*v*) glacial acetic acid.

For the second formulation, 100 mL of water, 10% (*w*/*w*) glycerol, 2% (*w*/*v*) cassava starch, and 1% (*w*/*v*) glacial acetic acid were mixed, stirred at 550 rpm, and heated to 67 °C for 20 min, adjusting the pH to 7 by adding sodium bicarbonate. This second formulation was slowly added to the first under stirring at 550 rpm. Twenty millilitres of this last mixture were taken and mixed with various concentrations of aniline. Finally, 20 mL of these formulations were placed in moulds and dried at 76 °C for 8 h to produce five films with the aniline concentrations and thicknesses specified in Figure 2 and Table 2. The films were stored in a desiccator (±25 °C, 50% RH) for 3 days until their subsequent characterisation. The same process described above was used to produce the films without blue aniline; see Figure 3. After discarding various formulations that produced films that could not be separated from the mould or that were excessively fragile, an optimal combination was chosen that included 2% (*w*/*v*) gellan gum, 2% (*w*/*v*) cassava starch, and 10% (*w*/*v*) glycerol. These percentages were modified after a bibliographic analysis of previous research on cassava starch, glycerol, and gellan gum, with the aim of differentiating the final film from others already described in the literature and optimising its characteristics.

#### 2.2.2. Measurement of Film Thickness Using a Micrometer and ASTM D6988-21 Standard

The thickness of the films was determined using a micrometer (Mitu-toyo 293-340-30, Kawasaki, Japan) in accordance with the standard method ASTM D6988-21 [26]. Measurements were taken at ten different points for the cassava starch and gellan gum films. This was carried out in three replicates. The results were expressed as the average of ten determinations (cm).

#### 2.2.3. Evaluation of the Optical Properties of Films (Colour Determination According to Food Standards)

The usual colour determination, according to food industry standards, was carried out for polymer films.

##### Characterisation of the Colour and Transparency of Films Using a Colorimeter (CIELab System)

A colorimeter (Konica Minolta CR-400, Tokyo, Japan) was used to evaluate the colour and transparency of the films. Square sections of each film (40 mm × 40 mm) were prepared, from which colour data were obtained at five different points per sample, performing three independent replicates. Before the measurements, the device was calibrated with a white reference plate (L = 93.7; a = 0.3159; b = 0.3324) and illuminant D65 (Konica Minolta CR-400, Tokyo, Japan) was used. The coordinates of the CIELab colour space were interpreted as follows: L* represents lightness (with values between 0 and 100), ±a* indicates a chromatic component that varies from green (−) to red (+), and ±b* indicates a chromatic component that varies from blue (−) to yellow (+). Finally, the colour of the films was expressed by the total colour difference (ΔE), calculated according to Equation (1) [27]:

(1) $$∆E=\sqrt{{\left({∆L}^{*}\right)}^{2}+{\left(∆a^{*}\right)}^{2}+{\left({∆b}^{*}\right)}^{2}}$$

where ΔE is the total colour difference and ΔL*, Δa*, Δb*—is the differential between the samples colour parameter and the colour parameter of the standard used as the film background. The transparency of the films (%T) was determined by considering L* = %T, assuming that a translucent film will generate the same brightness values (L*) as the white calibration plate (L* 0 = 100) and that any difference will be the result of a more opaque material (L* < 100) [28].

#### 2.2.4. Assessment of Water Solubility

Water solubility values were obtained using a method previously described by De Souza Falcão et al. [29]. Films with specific dimensions (2 cm × 2 cm) were placed in Erlenmeyer flasks and immersed in 30 mL of deionised water, maintaining slow agitation (30 rpm) in a Shaker incubator (Tecnal^®^ TE-520, Piracicaba, Brazil) at room temperature (25 °C).

After 24 h of immersion and stirring, the contents of the container were filtered, and the undissolved material was retained on filter paper to be dried in an oven at 105 °C for 24 h to obtain the final dry weight values according to Equation (2):

(2) $$Solubility=\frac{W_{i}-W_{f}}{W_{i}}\times 100$$

where *Wi* and *W_f_* are the initial and final weights of each sample, respectively. The results were expressed as water solubility percentage from independent triplicates of each film according to De Souza Falcão et al. [29].

#### 2.2.5. Moisture Content Measurement

The moisture content was determined by gravimetry, based on the AOAC Official Methods of Analysis (AOAC, 2005; method 930.15). For this, film samples were cut into rectangles (2 cm × 2 cm) and thermally dried at 105 °C in a hot air oven (MB25, OHAUS, Parsippany, NJ, USA) until constant mass. The moisture content (%) was calculated by dividing the loss of mass during drying by the initial weight of the sample [30].

#### 2.2.6. X-Ray Diffraction (XRD) Analysis and Quantitative Determination of the Relative Crystallinity Index

X-ray diffraction analysis (XRD) of cassava starch and gellan gum films without blue aniline was performed following the methodology of Grisales-Mejía et al. [31]. An X-ray diffractometer (Rigaku Miniflex 600, Tokio, Japón), operated at a voltage of 40 kV and a current of 30 mA with a copper objective, was used. Diffractograms were obtained within an angular range of 5–70° (2θ) at a scanning speed of 5°/min. The relative crystallinity index (%) of the samples was estimated quantitatively by calculating the ratio of the crystalline area to the total area of the diffractogram using OriginPro 2019b 64-bit software (OriginLab Corporation, Northampton, MA, USA).

#### 2.2.7. Determination of Thermal Diffusivity and Optical Absorption Coefficient Using Self-Normalised Photoacoustic Technique

A photoacoustic (PA) system was set up to determine the thermal and optical properties of polymer films with blue aniline in different concentrations. A detailed description of this system and the photoacoustic methodology can be found in another publication [32]; however, only a general description is provided here.

**Figure 4:**
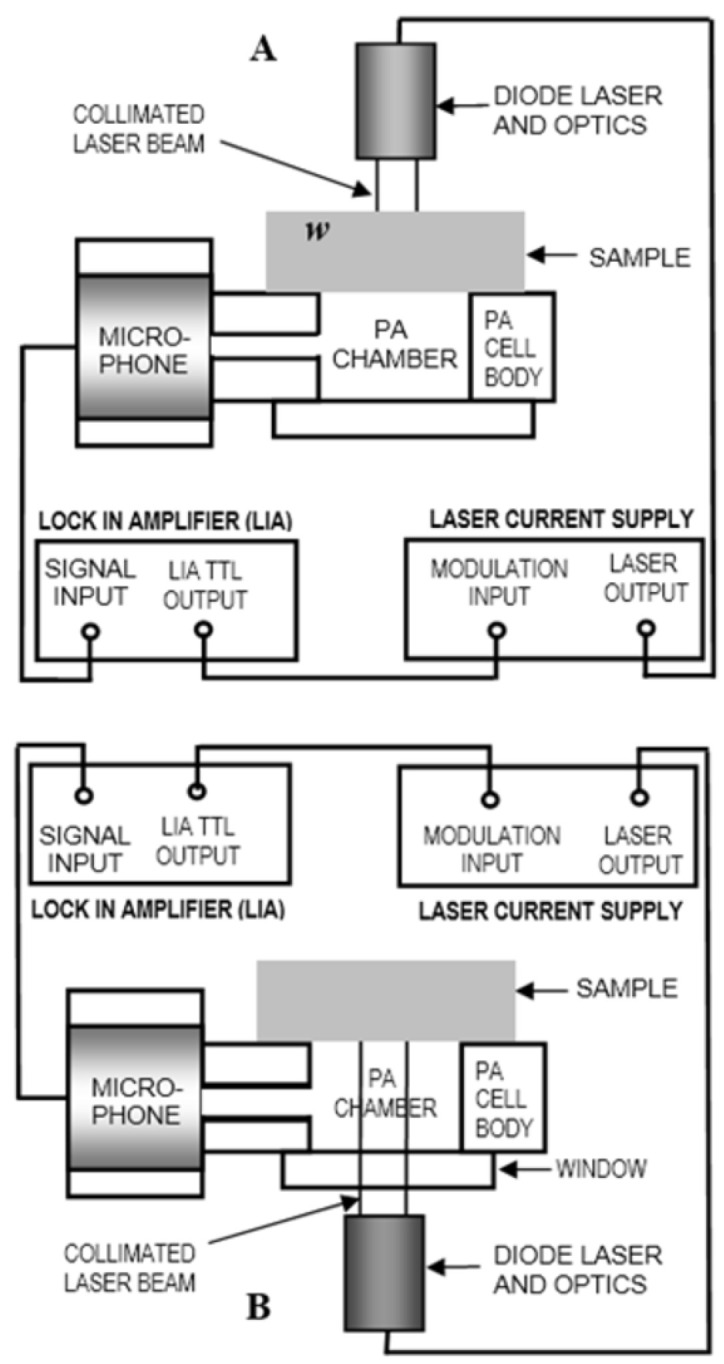
Cross section of the experimental photoacoustic (PA) setups for optical absorption coefficient measurements. (**A**) PA Transmission configuration. (**B**) PA Front configuration.

PA techniques are based on the conversion of radiation to sound by means of a PA cell (Figure 4). This cell consists of a cylindrical hole made on a metal body, communicate to a microphone through a thin channel (PA chamber). The PA chamber is sealed on one surface by means of the sample, and on the other side with an optical window. Absorption of modulated radiation and shinning the surface of a sample generates a modulated heat source; heat travels through the sample to reach the PA chamber where it generates pressure fluctuations (sound) at the modulated frequency. There are two possible PA configurations: transmission and front configurations. The transmission PA configuration is obtained when light shines on the sample’s surface opposite to the PA chamber (Figure 4A), whereas the front configurations is obtained when light shines the sample’s surface attached to the PA chamber (Figure 4B).

The typical PA experiment for thermo-optical characterisation consists of taking the PA signal as a function of the modulation frequency for both PA configurations and takes the self-normalised signal.

This latter signal consists of the ratio of PA signals, $\delta P_{T}\left(f\right)/\delta P_{F}\left(f\right)$, where $\delta P_{T}\left(f\right)$ stands for the PA transmission configuration while $\delta P_{F}\left(f\right)$ stands for the front configuration.

The experimental procedure consisted of taking a square section of approximately 2 cm on each side and placing it on the upper surface of the PA cell (see Figure 4). It is important to note that these were attached to the cell in this case without using vacuum grease, unlike the commonly used practice.

The various photoacoustic experiments in the transmission and frontal configurations (see Figure 4A and Figure 4B, respectively) were carried out by taking photothermal signals in amplitude and phase in a modulation frequency range of 3–31 Hz, taking frequency steps of 1 Hz. 

The analytical procedure was carried out on the self-normalised phase, which consists of the phase difference between the transmission and frontal PA configurations, in accordance with the analytical scheme reported by Balderas-López [32,33].

#### 2.2.8. Assessment of Biodegradation in the Natural Environment (Soil Burial Method)

The bioplastic films made from cassava starch and gellan gum without blue aniline, previously weighed and with a diameter of 2.85 cm, were dried in an oven at 70 °C for 24 h and then buried at a depth of 10 cm in the soil. To evaluate natural biodegradation under landfill conditions, the films were weighed every 3 days for a period of up to 15 days. At each measurement point, the samples were washed with running water to remove soil residues and then dried in an oven at 70 °C for 3 h to remove residual moisture. The biodegradability of the films was quantified by weight loss, following the method described by Nigam et al. [34] with some modifications. The experiments were performed in triplicate, and the standard deviation was calculated. Weight loss was calculated using Equation (3):

(3) $$\mathrm{Weight}\;\mathrm{loss}\;(\%)\;=\;\frac{W_{0}-W_{1}}{W_{0}}\times 100$$

where W_1_ is the initial dry weight of the bioplastic film and W_0_ is the final dry weight of the bioplastic film.

#### 2.2.9. Characterisation of Mechanical Properties (Puncture and Tension)

The mechanical properties of the conditioned films (cut into strips measuring 50 × 20 mm) were evaluated by means of puncture and tensile tests using a TA XT2i texture analyser (Texture Technologies Corp., Scarsdale, NY/Stable Micro Systems, Godalming, Surrey, UK) with a 5 kg load cell, following ASTM International standard methods (ASTM-D882:2010 [35]) similar to those described by Van Rooyen et al. [36] for all mechanical tests completed.

The puncture test used a 0.5 inch diameter metal ball tip, which acted on circular samples of 7.07 cm^2^ secured with clamps and screws to prevent slippage. 

The moving head descended perpendicularly at 0.80 mm/s; puncture resistance was recorded as the maximum load at fracture using Origin 2025b software.

Tensile strength, percentage elongation at break, and modulus of elasticity were determined using Origin 2025b software and a TA XT2i texture analyser equipped (Texture Technologies Corp., Scarsdale, NY/Stable Micro Systems, Godalming, Surrey, UK) with accessory tensile grips, with a test speed of 0.80 mm/s, a separation of 50 mm, and a test distance of 30 mm. Three films were tested per treatment. The different film thicknesses were measured on all conditioned films before completing the mechanical tests using a Mitutoyo digital micrometer [37]. These measurements characterised the strength and flexibility of the films [38].

#### 2.2.10. Statistical Analysis

The statistical analysis focused on the mean ± standard deviation of the results. One-way analysis of variance (ANOVA) was not used because only one experimental condition was evaluated (the cassava starch and gellan gum film with a constant concentration), which made it unnecessary to compare means between multiple independent groups. Therefore, the values presented are the average of three measurements obtained from identical replicates.

The main focus of the study was the development and characterisation of biodegradable films with a dye to validate the Beer–Lambert linear relationship in solids using photoacoustics, rather than the comparative evaluation of variations in film components with a control group.

## 3. Results and Discussion

Table 3 below shows the data obtained from the evaluations of thickness, moisture content, solubility, colour variation (ΔE), and relative crystallinity index (XRD) for the films studied in this manuscript, referring to the information published in the bibliography in the Results and Discussion section, which will be detailed later in this document.

### 3.1. Evaluation of the Physical and Optical Characteristics of Films (Thickness and Colour)

#### 3.1.1. Thickness

Selecting the ideal thickness for biodegradable films is essential and depends on the specific properties required for each application. A thinner film has transparency, flexibility, and elasticity, while a thicker film optimises protection by reducing moisture loss and permeability, which in turn provides greater resistance, although it reduces flexibility. Ultimately, thickness directly affects the efficiency of the protective barrier, which is crucial for the preservation, freshness, and final quality of products such as food [30].

The average thickness of the films in this study, with the addition of 2% (*w*/*v*) gellan gum, 10% (*w*/*w*) glycerol, 2% (*w*/*v*) cassava starch, and 1% (*w*/*v*) glacial acetic acid, was of 0.25 ± 0.02 mm. In another study, Lim et al. [39] combined only cassava starch and glycerol to produce a film with thickness of 0.19 ± 0.04 mm. The amount of each ingredient used (e.g., cassava starch and glycerol) showed a direct correlation with film thickness, as the concentration of these raw materials affects the viscosity of the suspensions. Thus, a higher amount of dry solids results in a thicker suspension.

Therefore, it was to be expected that the film obtained in this study would be thicker than that reported by Lim et al. [39], since it not only contained cassava starch, but also gellan gum, which increases the viscosity of the sample and the amount of solids compared to the reference sample, which only contained cassava starch.

On the other hand, the amylose content of cassava starch can increase the formation of hydrogen bonds due to the hydroxyl (–OH) groups present in the amylose structure, increasing the thickness of the film. As for the plasticiser, lower concentrations of glycerol resulted in thinner films when comparing the thickness values of the reference (0.19 mm) with those obtained in the present study (0.25 ± 0.02 mm) due to the greater binding capacity of the plasticiser to the starch matrix [41].

#### 3.1.2. Colour and Transparency

Table 4 shows the results with the colour parameters obtained by the CIELAB system and, as indicated, the cassava starch film presented an L* value of 92.07 ± 0.32, which indicates that it is almost white and transparent, according to Désiré et al. (2021) [42]. This is a satisfactory result since, being transparent, if it is used as a wound protector, it will allow for the wound closure to be verified without removing the dressing, as described by Nguyen et al. [43].

In contrast, the value of −0.40 ± 0.04 for a* in the same sample is associated with a green hue, while the value of 4.70 ± 0.10 indicates a yellow hue resulting from selective absorption of some wavelengths of the ingredients that make up the film, as reported by Moreno-Ricardo et al. [44]. This could be due to the drying temperature of the films and the fact that the gellan gum contained impurities such as traces of sugars and amino acids, which could have induced non-enzymatic browning reactions (Maillard reaction or caramelisation) that generate yellowish or brownish pigments [45].

In this study, a total colour difference (ΔE) of 5.57 ± 0.17 was obtained in the films (see Table 4). This value was higher than that reported by Velásquez-Castillo et al. [40] (ΔE* of 2.4–3.2) for cassava starch films containing quinoa starch nanocrystals.

The main cause of this greater colour difference in our films is attributed to the addition of gellan gum in their formulation. This incorporation generated a clearly perceptible colour change, manifested in the appearance of yellowish tones, as described in previous sections. The value obtained of ΔE = 5.57 confirms that this colour variation is clearly observable, since a total colour difference (ΔE) in the CIELAB system greater than 3 is generally considered perceptible to the human eye [46]. 

Considering the aforementioned values, the tone of the films analysed was extremely clear, almost white, and presented a slight and delicate yellowish tint. On the other hand, the presence of green colours was practically nil, indicating neutrality on that chromatic axis.

### 3.2. Determination of the Aqueous Solubility of Films

The ability to dissolve in water is one of the essential properties of a biodegradable material, influencing its level of water resistance. Overall, the film made from cassava, gellan gum, and glycerol was soluble in water (89.23 ± 1.03%). In contrast, the cassava starch film, which was modified by adding a bioactive compound, reported by De Souza Falcão et al. [29], showed a percentage of 35.60 ± 1.03%. The increase in the film’s solubility can be attributed to the incorporation of a water-soluble plasticiser, specifically glycerol, which is introduced into the intermolecular spaces of the polymer structure. This is due to its low molecular weight and low molar attraction constant, characteristics that allow for it to interrupt van der Waals interactions and hydrogen bonds between molecules, as well as reducing the overall rigidity of the polymer matrix [47].

Furthermore, it should be noted that the gellan gum added to the matrix, due to its hydroxyl groups, could help to reduce amylose-amylose, amylopectin-amylopectin, and amylose-amylopectin interactions in the starch, which can increase the number of hydrophilic groups available to interact with water [48]. The film produced showed high water solubility; however, this characteristic limits its application with liquids. Its main use would be focused on packaging food with low water activity or dry products, as well as single-use biodegradable disposable packaging. In this context, solubility helps to accelerate degradation in high-humidity environments or during composting, ensuring that, as single-use packaging, its environmental impact at the end of its life cycle is minimal.

### 3.3. Quantification of Moisture Content in Films

Food packaging and coatings must have low water activity in order to prevent the growth of pathogenic microorganisms and agents that cause surface deterioration, as well as to ensure product preservation [49]. In similar way as observed with thickness, it was determined that the amount of plasticiser significantly influenced moisture content. For the present study, a moisture value of 11.30 ± 0.28% was obtained, which is lower than that reported by Mueller et al. [15]: 14.33 ± 0.54%.

This was to be expected, since 8.4 g of cassava starch and 15.87 mL of glycerol were added to the reference films, while in the present study, 3 g of cassava starch and 1 mL of glycerol were used. Therefore, a higher concentration of glycerol increases the moisture content of the films. This is due to the hygroscopicity of glycerol, with hydroxyl groups in its molecular structure that can interact with water through hydrogen bonds. 

The adsorbed water molecules act as plasticisers, reaching the amorphous areas of the polymer network and promoting the plasticisation process in the films, an effect that is intensified with an increase in the relative humidity of the environment, as reported by Suh et al. [50]. From the above, it can be concluded that the film has a low moisture content compared to similar films. This characteristic makes it suitable for food coating, where low water activity is crucial to prevent the development of pathogenic and spoilage microorganisms on its surface.

### 3.4. Microstructural Characterisation by X-Ray Diffraction (XRD) and Determination of Relative Crystallinity

**Figure 5:**
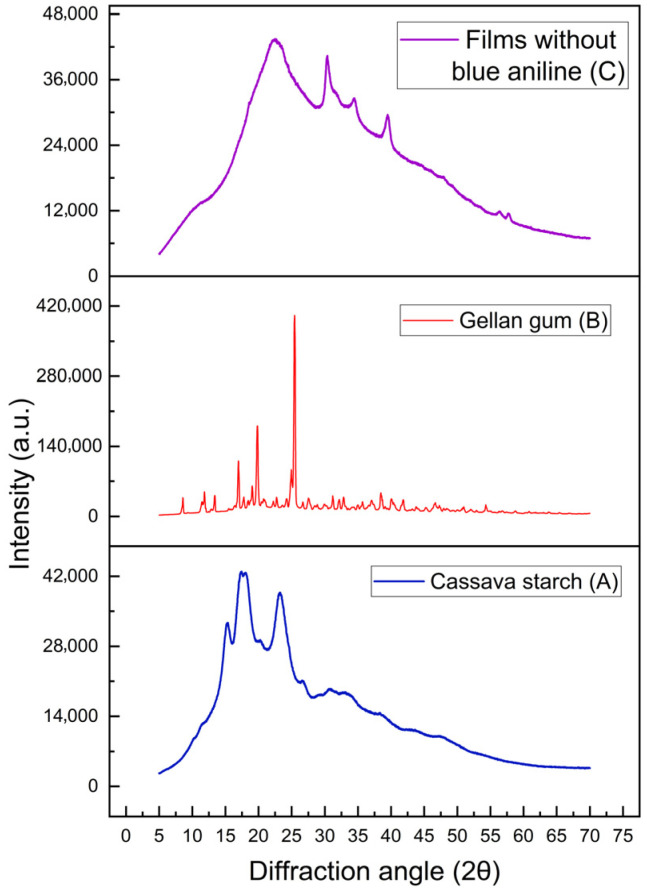
X-ray diffraction patterns of cassava starch film (**A**), gellan gum (**B**), and native cassava starch (**C**).

The polymer chains of gelatinised starch formed by recrystallisation or retrogradation are reorganised to form a starch film [51]. During this process, the surface structure of the film depends on the type and content of the plasticiser used. X-ray diffraction analysis was performed to investigate the degree of crystallinity of the developed film. Figure 5 shows the trends in the XRD patterns of cassava starch films with gellan gum, glycerol, glacial acetic acid, and sodium bicarbonate.

As shown by the XRD patterns of the film (Figure 5A), several peaks appeared at 22.22°, 30.38°, 34.1°, and 39.56°. The diffractogram of the films showed a broad peak around 22.22° at the 2θ scale, indicating their essential amorphous nature.

The characteristic peak observed around 30.38° is a clear indicator of crystallinity in the films. This phenomenon can be attributed to the presence of acetic acid and amylose, which function as a solvent and crosslinking agents, promoting the formation of crystalline zones. In addition, the self-organising capacity of long, linear amylose chains, together with the starch retrogradation process during the drying and storage of polymer films, promotes the reassociation of these chains into ordered structures. This process not only increases crystallinity, but also gives the film greater strength and rigidity [48].

The broad peaks at 34.1 and 39.56° indicate intermolecular interaction between gellan gum and cassava starch, becoming sharp and shifting to the right, possibly due to the interaction between starch chains. Figure 5C shows that glycerol reduces polymeric interactions, as noted by Donmez et al. [51].

On the other hand, compared to the XRD pattern of gellan gum (see Figure 5B), all its peaks disappear in the film pattern, indicating the alteration of the corresponding crystalline regions, as thermal processing gelatinised the gellan gum [52]. Furthermore, it should be noted that intense peaks at angles of 2θ = 19.06° and 22.02° are observed for pure gum, as reported by Naachiyar et al. [53]. However, as can be seen in the diffractogram, the gum presented other intense peaks at angles of 2θ = 16. 96°, 19.82°, and 25.44°, which showed a very pronounced increase in intensity and a decrease in amplitude, possibly due to impurities from the presence of salts, as the gum used was of molecular cooking grade. In addition, gellan gum is an anionic polysaccharide. This means that it has negative charges in its structure and, to maintain electrical neutrality, it must associate with cations (positively charged ions), which are components of salts. It is therefore also known as “pure sodium-type” gellan gum and may contain the following metal ions: Na^+^ (2.59%), K^+^ (0.009%), Ca^2+^ (0.02%), and Mg^2+^ (0.001%), as described by Annaka [54].

The film produced from cassava starch and gellan gum, plasticised with glycerol, had a crystallinity of 27.40 ± 1.68%, a value that is intermediate in relation to other biodegradable materials. In this regard, an analysis carried out by Tafa et al. [55] reported a higher crystallinity, reaching 34.18%, in films made from starch, while Nigam et al. [34] recorded a lower value of 16.38 ± 4.20% in a bioplastic based on plasticised cellulose acetate.

This balance in the structure, promoted by hydrogen bonds between gellan gum, glycerol, and cassava starch, provides ideal semicrystalline characteristics. Generally, a crystallinity range between 15% and 30% is considered optimal for films in contexts such as food packaging [56].

### 3.5. Thermo-Optical Properties of Blue Aniline Films Using the Self-Normalised Photoacoustic Technique

Thermo-optical characterisation of polymer films with blue aniline at different concentrations was carried out using a self-normalised photoacoustic technique. According to the applicable theoretical model [32], when sample absorbs light following the Beer–Lambert model, the self-normalised PA signal ($\delta P_{N}\left(f\right)$) is given by Equation (4):

(4) $$P_{N}\left(f\right)=\frac{\delta P_{T}\left(f\right)}{\delta P_{F}\left(f\right)}=\;\;=\frac{(1+b_{gm})(1+r)e^{-\beta l}e^{\sigma_{m}l}-2(r+b_{gm})-(1-b_{gm})(1-r)e^{-\beta l}e^{-\sigma_{m}l}}{(1+b_{gm})(1-r)e^{\sigma_{m}l}+2(r-b_{gm})e^{-\beta l}-(1-b_{gm})(1+r)e^{-\sigma_{m}l}}$$

where β is the sample’s optical absorption coefficient at the selected wavelength, $\sigma_{m}$ = (1 + i) ${\left(\pi f/\alpha_{m}\right)}^{1/2}$, $\alpha_{m}$ been the sample’s thermal diffusivity, $b_{js}$ = $e_{j}$/$e_{s}$ is the ratio of thermal effusivities, and r = β/$\sigma_{m}$. The reported analytical procedure for measuring optical absorption coefficients of solid samples involves a fitting procedure over the tangent of the self-normalised phase using a limiting expression obtained from Equation (4) [32]. This procedure yields the fitting parameter $P_{2}=\beta \sqrt{\frac{\alpha }{\pi }}$, from which the sample’s optical absorption coefficient can be obtained ones knowing its thermal diffusivity.

In this context, this thermal property must be measured independently; a way to conduct this involves the same PA self-normalised experimental setup, but assuming that the sample absorbs light in the surface absorption model. To ensure this, the optical limit sample is usually painted black, assuming that this procedure does not introduce a large variation in this thermal property of the original sample [32]. To overcome these inconveniences, in this work, the complete self-normalised phase from Equation (4) was fitted using MATLAB R2025b. The analytical procedure render in this case two fitted parameters, P2 and P3, from which a sample’s thermal diffusivity can be obtained according to $\alpha ={\left(\frac{l}{P2}\right)}^{2}\pi$ and $\beta =2\sqrt{\frac{\pi }{\alpha }}P3$.

**Figure 6:**
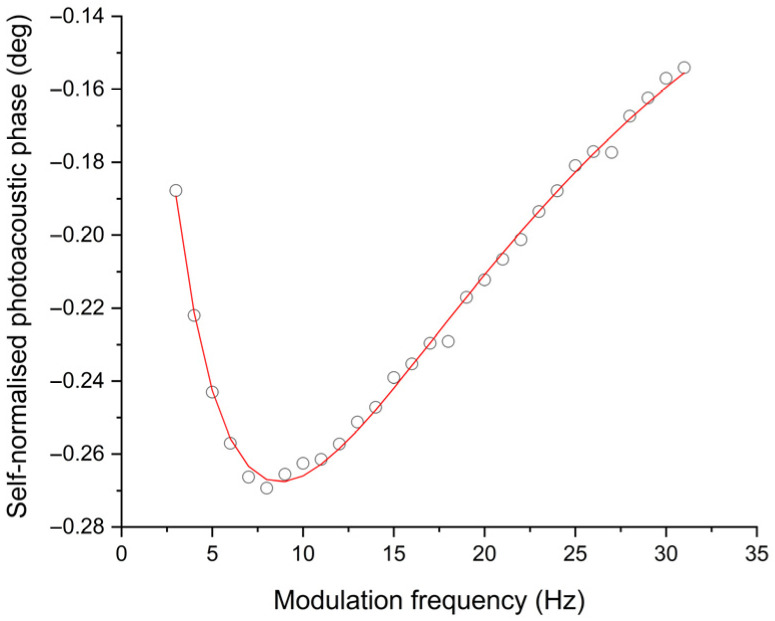
Self-normalised photoacoustic phase as a function of modulation frequency for polymer film at 0.64 mg of aniline blue/20 mL of polymer suspension (Sample 2.1, Table 2). Continuous line is the best fit to phase of Equation (4).

**Figure 7:**
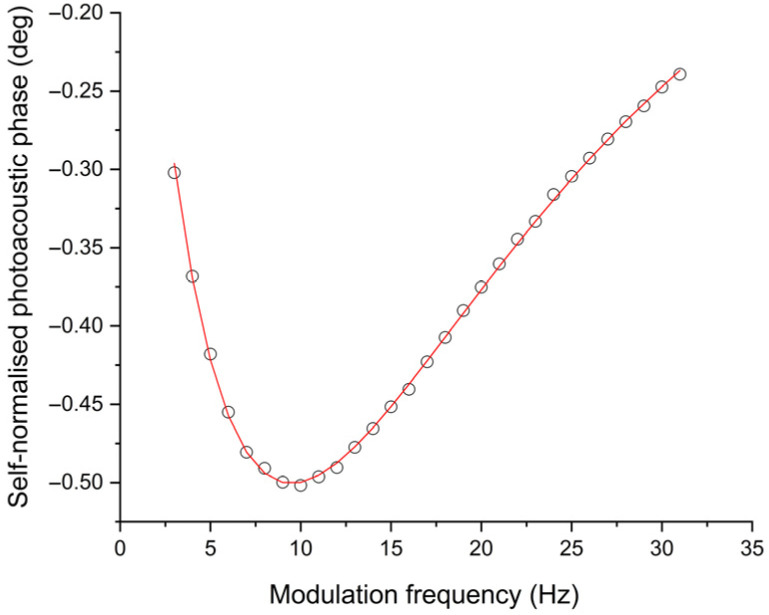
Self-normalised photoacoustic phase as a function of modulation frequency for polymer film at 1 mg of aniline blue/20 mL of polymer suspension (Sample 4.1, Table 2). Continuous line is the best fit to phase of Equation (4).

Figure 6 and Figure 7 show the self-normalised photoacoustic phase as a function of the modulation frequency for two samples, 2.1 and 4.1, respectively (see Table 2). Continuous lines in these two figures are the best fit to phase from Equation (4).

The corresponding values for thermal diffusivity and optical absorption coefficient (at 660 nm) for the five samples studied in this work are summarised in Table 5; uncertainties were estimated by means of the usual formulas for error propagation taking the error in parameters as the ones provided by the software. Small variations in terms of thermal diffusivity for these samples can be observed. This is an indication that the dye does not change the structure of the polymer films at large extend.

It is then possible to obtain the thermal diffusivity of the polymer films with this formulation as the average of these five thermal diffusivity values (Table 5, column 3), α= 0.0013 ± 0.0002 ${cm}^{2}/s$. This value is larger than other ones reported in the literature for other polymers. For example, it is higher than that indicated by Balderas-López [33] for thin acetate films, a completely amorphous material, which was recorded as 0.00041 ${cm}^{2}/s$. On the other hand, this value was lower than the 0.00205 ${cm}^{2}/s$ reported by Fernández-Olaya et al. [46] in films prepared with 0.8% (*w*/*v*) potato starch and 1% sorbitol.

The relatively high thermal diffusivity values observed in cassava starch and gellan gum films directly reflect their degree of crystallinity, as demonstrated by X-ray diffraction (Figure 5C). The increase in crystallinity may be associated with the intervention of acetic acid, as well as with the amylose chains present in cassava starch, which function as a solvent and crosslinking agent.

Their primary purpose is to facilitate the formation of hydrogen bonds between the hydroxyl groups of the starch chains, which in turn stabilises the organised sections of the polymer structure [48]. However, if a lower thermal diffusivity value is observed compared to the polymer reported by Fernández-Olaya et al. [24], this could be because glycerol, having a lower molecular weight than sorbitol, is able to intercalate between the polymer chains more easily, separating them and reducing the Van der Waals forces. This minimisation of polymer–polymer interactions leads to a more flexible and disorganised network than that obtained with sorbitol. In contrast, sorbitol appears to promote the formation of a more cohesive and stable biopolymer network through interactions that act as “bonding points” or “crosslinks” (albeit reversible or dynamic), resulting in a more uniform film with improved mechanical properties [24].

Thermal diffusivity is sensitive to the crystallinity of the polymer. An increase in crystallinity can increase thermal diffusivity, as crystalline regions have a more rigid molecular order and provide a more efficient pathway for heat transfer, in contrast to amorphous regions, which are more disordered and present obstacles to thermal conduction. Bento et al. [57] have published studies on the measurement of thermal diffusivity in different polymer films, such as PET, LDPE, PP, and epoxy resins, in which it has been observed that variables such as dyeing time and temperature in PET affect thermal diffusivity, indicating microstructural changes and changes in the internal organisation of the film. In this way, thermal diffusivity in polymer films not only provides thermal information about materials, but also allows for structural changes such as crystallinity levels or modifications induced by doping and manufacturing processes to be inferred [58].

**Figure 8:**
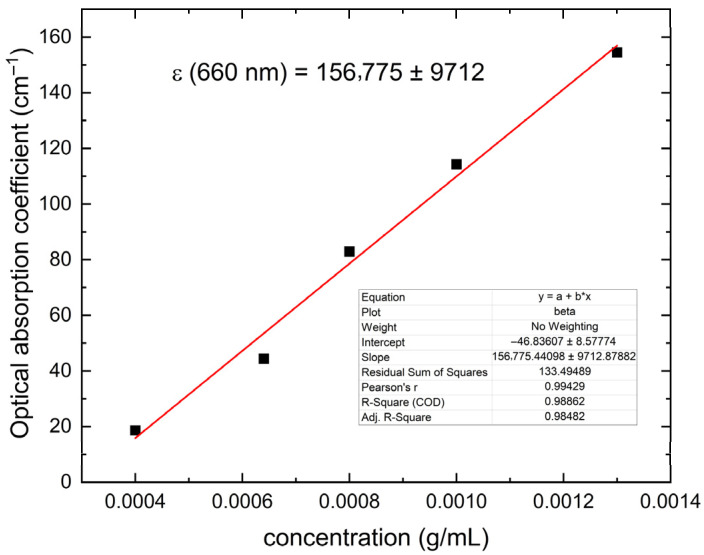
Optical absorption coefficient as a function of dye concentration for samples of cassava starch and gellan gum films. The straight line shows the range of dye concentrations where the Beer–Lambert model applies. The absorptivity shown in this graph corresponds to the slope of the best linear fit.

On the other hand, the optical absorption coefficients (Table 5, Column 4) show a very good linear correlation with the dye concentration, as can be seen in Figure 8. This demonstrates once again that Beer–Lambert’s law for light absorption applies in this case within the range of dye (0.0004–0.0013 g of dye inside mL of polymer solution) used in this work. The solid line in this figure is the best linear fit, and the slope is the absorptivity of the dye inside this polymer matrix. The value of this important optical property was in this case of $\varepsilon \;(660\;mn)\;=\;(156,775\pm 9712)\;{cm}^{-1}/$(g of blue aniline/mL of polymer suspension). 

It should be noted that this latter analysis is impossible to make using conventional spectroscopy given the high concentration of absorbing molecules that interact electrostatically, altering their absorption capacity and breaking the linearity within this solid matrix, which means that Beer–Lambert’s laws for light absorption do not apply to solid samples when using these techniques, which are based on the detection of transmitted light. For this reason, conventional spectroscopy is only useful for liquid samples with low dye concentrations.

All fitting procedures were performed using commercial software (MATLAB R2025b) using the standard error of the software’s fitted parameters as a measure of uncertainty.

For accurate photoacoustic modelling, three replicates per measurement were performed to compensate for thickness variation, which affects thermal diffusion length and optical absorption.

The main concern was to ensure that the film thickness exceeded the thermal diffusion length of the material, which required thicknesses greater than 100 microns due to the low diffusivity of the polymers. This parameter was controlled by adjusting the volume of the polymer solution poured into the mould when making the films for this technique.

### 3.6. Assessment of the Biodegradation of Films in the Natural Environment (Soil Burial Method)

**Figure 9:**
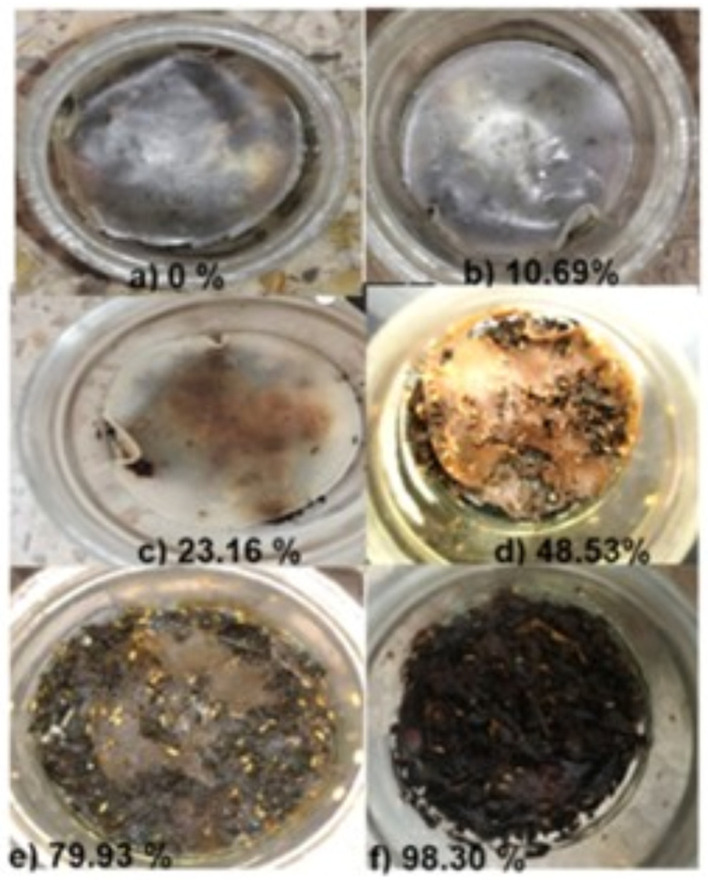
Biodegradability test. Photographic record of the degradation of films containing 2% (*w*/*v*) gellan gum, 10% (*w*/*w*) glycerol, 2% (*w*/*v*) cassava starch, and 1% (*w*/*v*) glacial acetic acid. (**a**) Film on day zero. (**b**) Film after 3 days. (**c**) Film after 6 days. (**d**) Film after 9 days. (**e**) Film after 12 days. (**f**) Film after 15 days.

Figure 9 illustrates the decomposition of films made from cassava starch and gellan gum produced in this research. It was found that all films showed their highest degradation rate in the first nine days. After this period, the films experienced a 48.53% loss in weight. Subsequently, the weight loss continued to increase, reaching a maximum of 98.30% in the films after 15 days (see Table 6). The data obtained suggest that the addition of glycerol to the films could facilitate degradation since, as discussed earlier in the results related to solubility, glycerol infiltrates the intermolecular spaces of the polymer structure. This interferes with van der Waals interactions and hydrogen bonds in the film, decreasing the overall rigidity of the polymer matrix. As a result, this increases its solubility in the presence of moisture and facilitates its hydrolysis by microorganisms present in the soil. The findings are consistent with those reported by Nigam et al. [34] and Kammoun et al. [59], who demonstrated that the inclusion of plasticisers in biopolymers and chitosan biofilms, respectively, increases the rate of biodegradability. Consequently, films produced from cassava starch and gellan gum proved to be biodegradable under natural soil conditions.

### 3.7. Analysis of the Mechanical Properties of Films (Puncture and Tension)

The key mechanical properties of biodegradable films include tensile strength, elongation at break, and Young’s modulus, which indicate the strength, elasticity, and stiffness of the material, respectively [37]. These characteristics determine the film’s application in packaging or as protective films in biomedicine and can be modified by additives or polymer combinations. Materials must be strong and flexible enough to withstand stress during manufacture and use. Evaluating these parameters is essential to ensure the viability and functionality of films, ensuring adequate protection and durability [56]. Therefore, the results obtained for the films in terms of puncture resistance, tensile strength, and elongation resistance tests will be described (see Table 7).

#### 3.7.1. Puncture Test

The films in this study with a formulation of 2% (*w*/*v*) gellan gum, 10% (*w*/*w*) glycerol, 2% (*w*/*v*) cassava starch, and 1% (*w*/*v*) glacial acetic acid showed a maximum average puncture force of 41 ± 0.05 N and a maximum displacement of 4.55 ± 0.08 mm. These results are higher than those reported by Tafa et al. [55] for similar materials, which were 15.02 N and 9.59 mm, respectively, as well as those obtained by Van Rooyen et al. [37], which were 31.75 ± 2.38 N and 4.04 ± 0.38 mm for other biodegradable films.

This difference can be attributed to the formation of hydrogen bonds between the hydroxyl groups of starch and glycerol for the films in this study, where glycerol molecules interfere with the interactions between starch macromolecules. This results in an increase in the elasticity of starch films. Also, the increase in the proportion of starch in the suspension used for film formation led to greater puncture resistance. According to Cortés-Rodríguez et al. [62], it was observed that the deformation of cassava starch films increased due to the greater flexibility of the polymers in the presence of glycerol.

**Figure 10:**
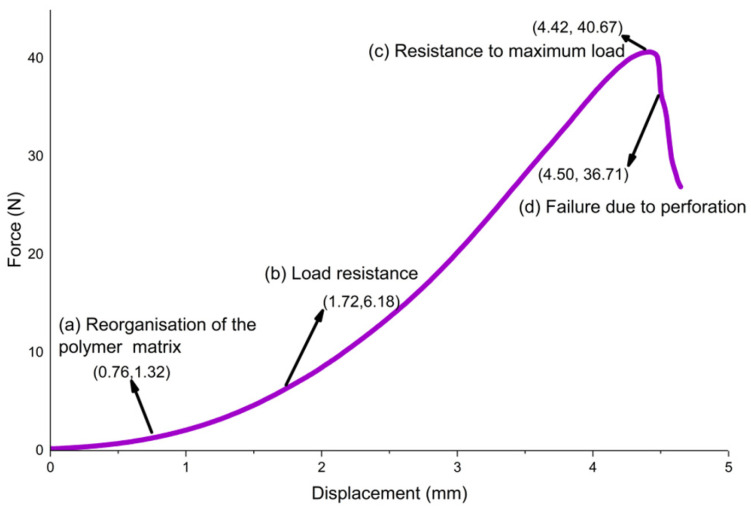
Failure mechanism of puncture resistance demonstrated by a load displacement curve for the films in this study with the addition of 2% (*w*/*v*) gellan gum, 10% (*w*/*w*) glycerol, 2% (*w*/*v*) cassava starch, and 1% (*w*/*v*) glacial acetic acid. The curve is the average of the results obtained from measuring three replicas of the bioplastic films.

On the other hand, Figure 10 shows a curve illustrating the failure due to the penetration resistance of the analysed films. This curve is divided into four stages: polymer matrix restructuring, load resistance, maximum resistance, and puncture failure, as reported by Dias Filho et al. [13] and Raimondo [63] for other materials. The curve begins with a slight slope when the indenter sphere begins to touch the elastic sample (see Figure 10a at coordinates (0.76, 1.32)). Since the polymer matrix still contains voids due to its amorphous nature (see Figure 5C related to XRD), this phenomenon is commonly recognised as a free volume of porosity at the microscopic level, which can be reorganised without difficulty without opposing the movement of the probe, as glycerol weakens the intermolecular bonds (especially van der Waals forces) within the polymer matrix and hydrogen bond-type intermolecular interactions (–OH) between biopolymers, such as gellan gum and hydrophilic polysaccharides (cassava starch), which act as reversible bonds between polymer chains, providing cohesion without forming rigid covalent bonds, resulting in greater flexibility and disorganisation of the polymer chains [48,60]. As the polymer chains lose their ability to move relative to each other, they begin to develop internal stresses in the material as the interaction between the chains in the polymer matrix increases [64].

Load resistance increases (see Figure 10b at coordinates (1.72, 6.18)) due to this interaction, resulting in a region of steeper slope.

Finally, the material develops new voids as the interaction between the polymer matrix chains fails, reaching a maximum load resistance (see Figure 10c at coordinates (4.42, 40.67)). When the pressure on the material exceeds the load that the interaction between the polymer matrix chains can withstand, the material breaks (see Figure 10d at coordinates (4.50, 36.71)).

#### 3.7.2. Tensile Test

The films in this study with the addition of 2% (*w*/*v*) gellan gum, 10% (*w*/*w*) glycerol, 2% (*w*/*v*) cassava starch, and 1% (*w*/*v*) glacial acetic acid had an elastic modulus of 55.54 ± 4.72 MPa, which is higher than that reported by Behera et al. [60] (40.9 ± 6.04 MPa). This larger elastic modulus is explained by the intense intermolecular interactions, mainly through hydrogen bonds, that are established between the hydroxyl groups of the starch and gellan gum, which generate an orderly and compact polymer network.

Although glycerol partially reduces these interactions by interposing itself between the chains, it also attenuates rigidity by enhancing flexibility, thus achieving an adequate balance between rigidity, mechanical strength, and structural stability in the film [65].

Hazrol et al. [61] showed that the elastic modulus of corn starch films decreases as the amount of glycerol increases, a phenomenon related to the appearance of discontinuities in the polymer matrix of the film once dry.

In this study, the films showed a tensile strength of 4.75 ± 0.38 MPa and an elongation at break of 19.19 ± 1.55%. The tensile strength value was higher than that reported by Hazrol et al. [61] for a corn starch film with 30% glycerol, which was 2.53 MPa. This suggests that, in the films in this study, the presence of bonds such as hydrogen bonds or other intermolecular interactions (e.g., Van der Waals forces) function as anchor points between the polymer chains. These bonds hinder slippage between chains when a tensile force is applied, which improves the transfer of the applied stress along the chains and increases the internal cohesion and structural stability of the material [66,67].

In addition to the elastic modulus and tensile strength, the film showed a percentage elongation at break of 19.19 ± 1.55%, higher than the 13.33 ± 0.30% reported by Narváez-Gómez et al. [22] for a film made with yam starch and 15% glycerol.

This increases in elongation are due to the hydrophilic nature of the polymer chains in the starch and gellan gum mixture, which reinforces intermolecular forces and improves the ability of molecular chains to move, facilitating greater extension. However, when glycerol levels are increased, elongation decreases because glycerol acts by decreasing hydrogen bond interactions between polymer molecules, as evidenced in the cited study [68].

Therefore, a film with low tensile strength (4.75 ± 0.38 MPa) was obtained because the plasticiser (glycerol) interferes with the interaction between the chains of the polymer matrix, facilitating their sliding and increasing flexibility. This occurs because glycerol decreases the rigidity of the network, generating a less ordered structure that allows for greater molecular movement, which resulted in a high percentage of elongation at break (19.19 ± 1.55%) and a high modulus of elasticity (55.54 ± 4.72 MPa) [69].

It should be noted that these films presented a stress–strain curve, which we will describe below.

**Figure 11:**
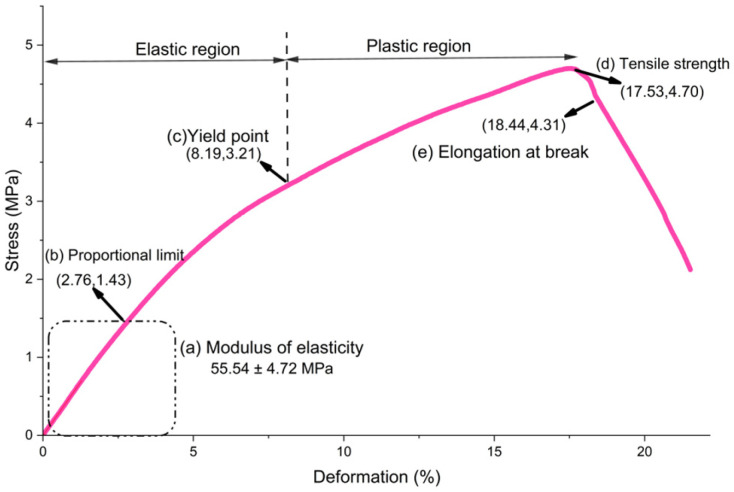
Stress–strain curve graph of the films in this study with the addition of 2% (*w*/*v*) gellan gum, 10% (*w*/*w*) glycerol, 2% (*w*/*v*) cassava starch, and 1% (*w*/*v*) glacial acetic acid. The curve is the average of the results obtained from measuring three replicas of the bioplastic films.

Figure 11 shows a typical stress–strain curve that describes how the applied stress varies with respect to the strain in the films analysed. This curve consists of two main zones: elastic and plastic. In the elastic region, which extends to the elastic limit or proportional limit, the deformation is reversible. In this initial stage, the stress–strain relationship is linear and follows Hooke’s law, where the slope represents the modulus of elasticity (Figure 11a). As it approaches the proportional limit, the slope decreases and finally reaches zero at the elastic limit (see Figure 11b at coordinates (2.76, 1.43)), at which point the applied stress is the creep resistance (see Figure 11c at coordinates (8.19, 3.21)). This indicates the onset of permanent deformation, as the material no longer recovers its original shape. Beyond this point, the material enters the plastic phase. First, the actual stress decreases due to strain softening, associated with the internal reorganisations of the material, until it reaches the tensile strength (see Figure 11d at coordinates (17.53, 4.70), which is the maximum stress it can withstand before fracturing. 

Subsequently, the orientation and alignment of the polymer chains cause strain hardening, where the stress increases again until final rupture (see Figure 11e at coordinates (18.44, 4.31)).

These behaviours reflect the complex mechanical properties of biodegradable films and allow us to understand their ability to withstand forces and deform under different conditions [67,70].

## 4. Conclusions

Biodegradable films were successfully produced by integrating various concentrations of cassava starch and gellan gum, as well as by incorporating blue aniline. The inclusion of the plasticiser had a notable impact on the physical, optical, microstructural, and mechanical characteristics of the films. These were found to be thin, semi-crystalline, and transparent, with high solubility, flexible, with low tensile strength, and a high modulus of elasticity, making them ideal candidates for the manufacture of plastics designed for single use before being discarded. In addition, a photoacoustic technique was used to the simultaneous measure the optical absorption coefficient (at 660 nm) and thermal diffusivity of these polymer films at different concentrations of blue aniline, demonstrating that this method is effective and non-destructive for thermo-optical characterisation of the samples. For the first time, it was possible to determine the absorptivity of the dye within the polymer matrix for this type of material, a property that cannot be obtained with conventional methods.

This technique allowed for quantitative optical characterisation and can be applied to other biodegradable or non-biodegradable polymer films, with different dyes and wavelengths, provided that an appropriate wavelength is used. The potential application of this photoacoustic technique for quantitative analysis of dyes inside solid matrix was proved. The biodegradability analysis confirms the ecological nature of the films under natural conditions. The maximum degradation rate was observed within 15 days. Consequently, the properties demonstrated by the films when the optimum amount of their components is added highlight their potential for the production of sustainable plastics. Cassava starch and gellan gum films, despite being biodegradable, have significant limitations: their high solubility (≈89.23%) makes them very sensitive to moisture, which compromises their structural integrity. Their low tensile strength and limited mechanical durability restrict them to single-use applications, preventing their use in packaging or protective materials that require robustness and prolonged water resistance.

## Figures and Tables

**Table 1:** Properties of biodegradable plastics (The data used to create the table have been adapted from Kumar et al [3]).

Properties	PLA	PHA	PBS	Starch
Synthesis methods	Chemistry	Microbial	Chemistry	Chemistry
Resistance to attraction (Mpa)	53–70	15–40	34	1–3
Melting point (°C)	120–170	160–175	115–118	72.3
Elongation at break (%)	10–100	1–15	560	47–51
Vapour transmission rate $\left(\frac{g}{m^{2}}\cdot día\right)$	15.94	2.36	83.8	16.2 ± 3.4
Oxygen transmission rate $\left(\frac{{cm}^{3}}{m^{2}}\cdot día\right)$	209.9	55.12	737.77	5.50 ± 0.531

PLA (polylactic acid). PHA (polyhydroxyalkanoate). PBS (polybutylene succinate).

**Table 2:** Aniline concentrations used in cassava starch and gellan gum films.

Label of the Film	Dye Concentration (g Aniline Blue/mL Polymer)	Film Thickness (cm) (±0.01)
1.1	4.0 × 10^−4^	0.0136
2.1	6.4 × 10^−4^	0.0193
3.1	8.0 × 10^−4^	0.0151
4.1	1.0 × 10^−3^	0.0146
5.1	1.3 × 10^−3^	0.0159

The values are the mean ± standard deviation of three independent replicates of polymer films containing 2% (*w*/*v*) gellan gum, 10% (*w*/*w*) glycerol, 2% (*w*/*v*) cassava starch, and 1% (*w*/*v*) glacial acetic acid with different concentrations of blue aniline.

**Table 3:** Comparative table of physical, optical, and structural properties of films.

Property Assessed	Value Obtained in This Study (Cassava/Gellan Gum Film)	Value in Cited Literature (Similar Material)	Attribution of Literature
Thickness	0.25 ± 0.02 mm	0.19 ± 0.04 mm	Lim et al. [39]
Colour (ΔE)	5.57 ± 0.17	2.4–3.2	Velásquez-Castillo et al. [40]
Solubility in water	89.23 ± 1.03%	35.60 ± 1.03%	De Souza Falcão et al. [29]
Humidity	11.30 ± 0.28%	14.33 ± 0.54%	Mueller et al. [15]
Relative crystallinity	27.40 ± 1.68%	16.38 ± 4.20%	Nigam et al. [34]

The values are the mean ± standard deviation of three independent replicates of polymer films containing 2% (*w*/*v*) gellan gum, 10% (*w*/*w*) glycerol, 2% (*w*/*v*) cassava starch, and 1% (*w*/*v*) glacial acetic acid.

**Table 4:** Colour test results.

Property to Be Analysed	Value Obtained in This Study
a*	−0.40 ± 0.04
b*	4.70 ± 0.10
L*	92.07 ± 0.32
ΔL	4.29 ± 0.13
Δa	−0.28 ± 0.04
Δb	3.54 ± 0.10
ΔE	5.57 ± 0.17

The values are the mean ± standard deviation of three independent replicates of polymer films containing 2% (*w*/*v*) gellan gum, 10% (*w*/*w*) glycerol, 2% (*w*/*v*) cassava starch, and 1% (*w*/*v*) glacial acetic acid.

**Table 5:** Thermal diffusivities (α) and optical absorption coefficients (β) at 660 nm for the polymer samples with blue aniline studies in this work (Figure 2) by means of the analysis of the self-normalised phase from Equation (4).

Dye Concentration (g Blue Aniline/mL Polymer Suspension)	Film Thickness (cm) (±0.01)	α $\;\left(\frac{{cm}^{2}}{s}\right)$	β $\left({cm}^{-1}\right)$
4.0 × 10^−4^ 6.4 × 10^−4^ 8.0 × 10^−4^ 1.0 × 10^−3^ 1.3 × 10^−3^	0.0136 0.0193 0.0151 0.0146 0.0159	0.0009 ± 0.0001 0.0016 ± 0.0002 0.0013 ± 0.0002 0.0013 ± 0.0002 0.0013 ± 0.0001	18.7 ± 4.5 44.4 ± 5.3 82.92 ± 10.7 114.3 ± 17.8 154.6 ± 13.8

The thickness values are the mean ± standard deviation of three independent replicates of polymer films containing 2% (*w*/*v*) gellan gum, 10% (*w*/*w*) glycerol, 2% (*w*/*v*) cassava starch, and 1% (*w*/*v*) glacial acetic acid with different concentrations of blue aniline. The thermal diffusivity and optical absorption coefficient values are the standard error.

**Table 6:** Biodegradability results for cassava starch and gellan gum films.

Time Period (Days)	Average Weight Loss (%) of Films in Triplicate
3	10.69 ± 0.35
6	23.16 ± 0.74
9	48.53 ± 1.58
12	79.93 ± 1.54
15	98.30 ± 1.01

The values are the mean ± standard deviation of three independent replicates of polymer films containing 2% (*w*/*v*) gellan gum, 10% (*w*/*w*) glycerol, 2% (*w*/*v*) cassava starch, and 1% (*w*/*v*) glacial acetic acid.

**Table 7:** Comparison of the mechanical properties (puncture, tensile strength, and elasticity) of cassava starch and gellan gum films with reference materials.

Mechanical Properties	Value Obtained in This Study (Cassava/Gellan Gum Film)	Value in Cited the Literature (Similar Material)	Literature Attribution
Puncture: Maximum Force	41 ± 0.05 N	15.02 N	Tafa et al. [55]
		31.75 ± 2.38 N	Van Rooyen et al. [37]
Puncture: Maximum displacement	4.55 ± 0.08 mm	9.59 mm	Tafa et al. [55]
		4.04 ± 0.38 mm	Van Rooyen et al. [37]
Modulus of elasticity	55.54 ± 4.72 MPa	40.9 ± 6.04 MPa	Behera et al. [60]
Tensile strength	4.75 ± 0.38 MPa	2.53 MPa	Hazrol et al. [61]
Elongation at break (%)	19.19 ± 1.55%	13.33 ± 0.30%	Narváez-Gómez et al. [22]

The values are the mean ± standard deviation of three independent replicates of polymer films containing 2% (*w*/*v*) gellan gum, 10% (*w*/*w*) glycerol, 2% (*w*/*v*) cassava starch, and 1% (*w*/*v*) glacial acetic acid.

## Data Availability

The original contributions presented in this study are included in the article. Further inquiries can be directed to the corresponding authors.
